# UBE2T promotes autophagy via the p53/AMPK/mTOR signaling pathway in lung adenocarcinoma

**DOI:** 10.1186/s12967-021-03056-1

**Published:** 2021-08-30

**Authors:** Jinhong Zhu, Haijiao Ao, Mingdong Liu, Kui Cao, Jianqun Ma

**Affiliations:** 1 Department of Clinical Laboratory, Biobank, Harbin Medical University Cancer Hospital, 150 Haping Road, Harbin, 150040 Heilongjiang China; 2 Department of Clinical Oncology, Harbin Medical University Cancer Hospital, 150 Haping Road, Harbin, 150040 Heilongjiang China; 3 Department of Thoracic Surgery, Harbin Medical University Cancer Hospital, 150 Haping Road, Harbin, 150040 Heilongjiang China

**Keywords:** UBE2T, Lung cancer, p53, Autophagy, Prognostic signature

## Abstract

**Background:**

Ubiquitin-conjugating enzyme E2T (UBE2T) acts as an oncogene in various types of cancer. However, the mechanisms behind its oncogenic role remain unclear in lung cancer. This study aims to explore the function and clinical relevance of UBE2T in lung cancer.

**Methods:**

Lentiviral vectors were used to mediate UBE2T depletion or overexpress UBE2T in lung cancer cells. CCK8 analysis and western blotting were performed to investigate the effects of UBE2T on proliferation, autophagy, and relevant signaling pathways. To exploit the clinical significance of UBE2T, we performed immunohistochemistry staining with an anti-UBE2T antibody on 131 NSCLC samples. Moreover, we downloaded the human lung adenocarcinoma (LUAD) dataset from The Cancer Atlas Project (TCGA). Lasso Cox regression model was adopted to establish a prognostic model with UBE2T-correlated autophagy genes.

**Results:**

We found that UBE2T stimulated proliferation and autophagy, and silencing this gene abolished autophagy in lung cancer cells. As suggested by Gene set enrichment analysis, we observed that UBE2T downregulated p53 levels in A549 cells and vice versa. Blockade of p53 counteracted the inhibitory effects of UBE2T depletion on autophagy. Meanwhile, the AMPK/mTOR signaling pathway was activated during UBE2T-mediated autophagy, suggesting that UBE2T promotes autophagy via the p53/AMPK/mTOR pathway. Interestingly, UBE2T overexpression increased cisplatin-trigged autophagy and led to cisplatin resistance of A549 cells, whereas inhibiting autophagy reversed drug resistance. However, no association was observed between UEB2T and overall survival in a population of 131 resectable NSCLC patients. Therefore, we developed and validated a multiple gene signature by considering UBE2T and its relevance in autophagy in lung cancer. The risk score derived from the prognostic signature significantly stratified LUAD patients into low- and high-risk groups with different overall survival. The risk score might independently predict prognosis. Interestingly, nomogram and decision curve analysis demonstrated that the signature’s prognostic accuracy culminated while combined with clinical features. Finally, the risk score showed great potential in predicting clinical chemosensitivity.

**Conclusions:**

We found that UBE2T upregulates autophagy in NSCLC cells by activating the p53/AMPK/mTOR signaling pathway. The clinical predicting ability of UBE2T in LUAD can be improved by considering the autophagy-regulatory role of UBE2T.

**Supplementary Information:**

The online version contains supplementary material available at 10.1186/s12967-021-03056-1.

## Introduction

Lung cancer is the primary origin of cancer-related mortality worldwide, with non-small-cell lung cancer (NSCLC) as the most predominant histological subtype of the disease [1, 2]. Over past decades, with oncogenic driver mutations recognized in NSCLC, along with the clinical application of an increasing number of inhibitors targeting these druggable mutations, patients have been offered a remarkable opportunity to elongate their lifespan [3]. Unfortunately, despite these molecular advances, a quite proportion of NSCLC remains incurable, mainly due to the advanced stage at diagnosis and therapeutic resistance [4, 5]. NSCLC is a heterogeneous disease, especially regarding molecular basis [3]. Therefore, studying and exhausting its biology are essential for the development of early detection biomarkers and effective therapies.

NSCLC is a complex disease. Numerous changes in many cellular processes are prerequisites to meet the demanding needs of tumor cell growth and survive in an established tumor, such as dysregulated angiogenesis, upregulated glycolysis, and immune evasion. In particular, autophagy is a catabolic process supplying energy and macromolecular precursors (amino acids, nucleic acids, sugars, and fatty acids) in normal cells [6]. Recently, it has become apparent that autophagy contributes to the growth and survival of various tumors, including lung cancer, by sustaining tumor metabolism and eliminating damaged organelles [7]. Therapies manipulating autophagy in cancer are underway in clinical trials, but the mechanism behind elevated autophagy in cancer warrants intensive investigations [6, 8].

Ubiquitin-conjugating enzyme E2T (UBE2T; also known as HSPC150) is a typical UBC domain-containing E2, which docks the RING finger or HECT domain of an E3 and facilitate the mono- or polyubiquitination of substrates. UBE2T was initially identified as an E2 in the Fanconi anemia (FA) pathway [9]. The binding of UBE2T to FANCL facilitates the monoubiquitination of FANCD2, which, in turn, enables the formation of a nuclear FA protein complex and activates DNA repair [9]. UBE2T depletion impairs DNA repair ability and causes chromosome abnormality, a feature of Fanconi anemia [9]. Besides its relevance in FA, accumulating evidence indicates that UBE2T also plays an oncogenic role in various cancer, including hepatocellular carcinoma [10], gastric cancer [11], breast cancer [12], multiple myeloma [13], osteosarcoma [14], and NSCLC [15]. Several studies have suggested the important implications of UBE2T in NSCLC [15–17]. UBE2T is significantly associated with poor prognosis in lung carcinoma [16, 17]. Moreover, UBE2T was found to stimulate NSCLC cell proliferation, migration, invasion, and epithelial–mesenchymal transition (EMT) [15]. Despite these intriguing findings, the functions of UBE2T remain largely unknown in the NSCLC carcinogenesis.

In this study, we explored whether UBE2T participates in the regulation of autophagy in the NSCLC cells and underpinning mechanisms. We found that UBE2T enhanced autophagy in NSCLCs by modulating subcellular localization and p53 and activating the p53/AMPK/mTOR signaling pathway. Moreover, the multi-gene signature integrating UBE2T and autophagy genes could robustly predict prognosis and drug sensitivity in NSCLC.

## Materials and Methods

### Cell culture

Human lung bronchial epithelial (Beas-2B) and a collection of lung cancer cells (A549, HCC827, NCI-H460, NCI-H1299, NCI-H1915, and H1650) were purchased from the Cell Bank of the Chinese Academy of Science (Shanghai, China). Cells were cultured in the DEME (Beas-2B), or RPMI 1640 (A549/A549cisR, HCC827/827GR, NCI-H460, H1299, H1915, and H1650 cells) medium (Gibco, Invitrogen) supplemented with 10% fetal bovine serum (PAN, Germany), penicillin G (100 U/ml, Beyotime, China), streptomycin (100 μg/ml, Corning, China) in a humidified incubator with 5% CO_2_, at 37 °C. Cisplatin-resistant A549 (A549cisR) and gefitinib-resistant HCC827 (827GR) were established in the laboratory. Briefly, to establish A549cisR, parental A549 cells were maintained in a culture medium with increasing concentrations of cisplatin (0.5–10 μg/ml; Sigma Aldrich; Merck KGaA) for more than 6 months. Besides, parental HCC827 cells were maintained in a culture medium supplemented with gradually increasing gefitinib from 0.1 to 10 μmol/l for 6 months to develop 827GR cells.

### Establishment of stable cell lines

Lentiviral vectors were purchased from GenePharma (Shanghai, China), including lentiviral UBE2T, empty vector, shUBE2T, and shControl (shCtrl). Three shRNA sequences targeting UBE2T were listed as follows: 5′-TTGTCTGGATGTTCTCAAATT-3′; 5′-GCTGCTCATGTCAGAACCCAA-3′; 5′-TGACATATCCTCAGAATTTAA-3′. After being infected with different Lentiviral vectors above, shUBE2T cells, control shRNA cells, UBE2T overexpression cells, and vector control cells were maintained in a culture medium with the addition of puromycin. Finally, western blotting was used to evaluate the levels of UBE2T in each type of cell.

### Proliferation assay

For cell proliferation assay, a Cell Counting Kit-8 (CCK8, MedChemExpress LLC) was adopted according to the manufacturer’s instructions.

### Cell starvation

When cells reached 80% confluence, the normal culture medium was replaced with EBSS. Cells were cultured in EBSS without fetal bovine serum for 8 h to induce autophagy. Afterward, cells were collected in a lysis buffer for western blotting analysis.

### Western blotting analysis

Firstly, the shUBE2T and shCtrl cells were treated with EBSS (Boster Bio-engineering Limited Co, Wuhan, China) or EBSS accompanied by pifithrin-α (PFT-α) (Selleck Chemicals, CA, USA), while the UBE2T and vector cells were handled with as indicated. Then, whole-cell protein extracted from those cells were lysed with a RIPA buffer (Beyotime, Wuhan, China) containing protease inhibitor mix (GE Healthcare, Piscataway, USA) and phosphatase inhibitor cocktail (Thermo, Rockford, IL, USA). And proteins were quantified using a BCA protein assay kit (Boster Bio-engineering Limited Co, Wuhan, China) according to the manufacturer’s instructions. A NE-PER™ nuclear cytoplasm fraction kit (Thermo Fisher Scientific) was used to separate nuclear proteins from cytoplasmic proteins.

Equal amounts of cell lysate were electrophoresed in SDS-PAGE (10% or 15%) and transferred to PVDF membranes. After blocking with 5% skimmed milk in TBST at room temperature for 1 h, the membranes were incubated overnight at 4 °C with primary antibodies against UBE2T (1:1000, Abcam), LC3II/I (1:1000, Abcam, Cambridge, CB2 0AX, UK), p62 (1:1000, Abcam), ATG5 (1:1000, Abcam), Beclin-1(1:1000, Abcam), p53 (1:1000, Cell Signaling Technology, Danvers, MA), AMPK (1:1000, Cell Signaling Technology), P-AMPK (1:1000, Abcam), mTOR (1:1000, Cell Signaling Technology), p-mTOR (1:1000, Cell Signaling Technology), β-actin (1:1000, Beijing Zhongshan Golden Bridge Biotechnology Co.Ltd, China), Lamin B1 (1:1000, Proteintech, Rosemont, IL) and GAPDH (1:80,000, Proteintech). After washing with TBST, the membranes were incubated with a secondary antibody (1:10,000, Beijing Zhongshan Golden Bridge Biotechnology Co. Ltd, China) for 1 h at room temperature. Finally, an ECL detection system was used to detect targeted protein bands. GAPDH was used as internal controls for western blotting.

### Data acquisition

The whole-transcriptome data (mRNA SeqV2) and clinical characteristics of human lung carcinoma were acquired from The Cancer Genome Atlas (TCGA) project (https://portal.gdc.cancer.gov/). The raw data of the tumor and normal tissues were processed by the “DESeq2” and “edgeR” package using R software (version 4.0.0).

### Survival analysis for UBE2T

We evaluate the association of UBE2T with patient survival in two GEO-LUAD cohorts, GSE13213 (n = 117) and GSE31210 (n = 205). Survival analyses were performed using an online tool, PrgnosScan (www.prognoscan.org).

### Patients and samples

We retrospectively collected formalin-fixed paraffin-embedded tissue specimens from 131 patients with NSCLC receiving surgical operation in the Harbin medical university cancer hospital from March 2013 to June 2013. These patients did not receive any anticancer treatments prior to surgery, and their clinical data were acquired from electronic medical records. Characteristics of patients were shown in Additional file 1: Table S1. All patients were followed up for more than 5 years from the day of the surgical operation. The approval of this study was obtained from the Ethics Committee of Harbin Medical University Cancer Hospital, and patients or relatives provided written consent before patients were enrolled in this study. The TNM Classification of Malignant Tumors (TNM) of patients with NSCLC was determined based on guidelines by the American Joint Committee on Cancer (AJCC) and the International Union Against Cancer (UICC) (2017).

### Immunohistochemistry (IHC) staining

A paraffin slicing machine (Leica, Germany) was used to cut paraffin-embedded samples into 4 μm slices. IHC staining was performed following the standard protocol as previously described [18]. After initial deparaffinization, antigen retrieval, and blockade, the anti-UBE2T antibody (cat#: ab154022, Abcam, Cambridge, MA, United States) was applied to sections overnight at 4 °C in a humidified container. A horseradish peroxidase-labeled secondary antibody (cat#: ab205718, Abcam, Cambridge, MA, United States) followed the next day. DAB (50:1, Novus Biologicals, Centennial, CO, United States) was used to visualize UBE2T staining, followed by hematoxylin counterstain. Sections skipping the primary antibody were used as the negative control. Each sample was designated a score of 0 (negative), 1 (mild), 2 (moderate), or 3 (strong) based on the relative staining intensity. Tissue with score ≤ 1 or ≥ 2 was defined as low and high expression, respectively.

### Gene set enrichment analysis (GSEA)

We downloaded the RNAseq data of 119 NSCLC cancer cell lines from the Cancer Cell Line Encyclopedia (CCLE) database (https://portals.broadinstitute.org/ccle/). This website contains the detailed expression data of 84,433 genes of 1457 cell lines. The CCLE project was initiated and developed by the Broad Institute, the Novartis Institutes for Biomedical Research, and its Genomics Institute of the Novartis Research Foundation. After data processing, we used the median expression level of the *UBE2T* gene acted as a cutoff value to dichotomize all NSCLC cell lines into *UBE2T*^high^ and *UBE2T*^low^ groups. GSEA software 4.0.0 was downloaded to analyze the UBE2T-associated signaling pathways (http://software.broadinstitute.org/gsea/index.jsp).

### Development of a prognostic signature with UBE2T-correlated autophagy genes

First, we obtained 24,250 UBE2T correlated genes (*P* < 0.05) in the TCGA-LUAD cohort. Second, a total of 232 autophagy-related genes were downloaded from the Human Autophagy Database (http://autophagy.lu/clustering/index.html). By intersecting UBE2T correlated and autophagy-related genes, we obtained 163 UBE2T-correlated autophagy genes. The following univariate Cox regression analysis yielded 36 genes associated with overall survival in LUAD. The least absolute shrinkage and selection operator (LASSO) Cox regression algorithm was used to build an optimal prognostic gene signature with the UBE2T correlated, and autophagy-related genes as previously described [19, 20].

This signature allowed us to quantify the risk of individual patients using the risk score, which was derived by integrating the expression and survival contribution of each included gene. We also construct a nomogram to validate the prognostic accuracy of the gene signature alone or in combination with clinical characteristics, using the survival and the rms package for R. Decision curve analysis was also performed.

### Prediction of chemosensitivity

To interrogate whether this UBE2T-correlated autophagy gene signature is related to chemosensitivity in LUAD, we computed the half inhibitor concentration (IC50) of standard chemotherapeutic drugs using the pRRophetic [21, 22]. This R package contains an algorithm that may predict clinical chemotherapeutic response by calculating tumor gene expression levels. This study assessed IC50 for paclitaxel, gemcitabine, cisplatin, docetaxel, gefitinib, and erlotinib.

### Statistical analysis

Statistical analysis was carried out using the SPSS version 22 (SPSS, Inc., Chicago, IL, USA). The experimental results in vitro and in vivo were presented as the mean ± standard deviation (SD). The student’s t-test was used to analyze differences between two groups. For comparisons between multiple groups, one-way or two-way analysis of variance (ANOVA) was performed, followed by Student-Neuman–Keuls (SNK) tests in order to achieve means separation. Differences were considered statistically significant at *P* < 0.05. R software (version 4.0.0; https://www.r-project.org/) was used for bioinformatic analysis.

## Results

### The expressional level of UBE2T in different lung cancer cell lines

**Fig. 1:**
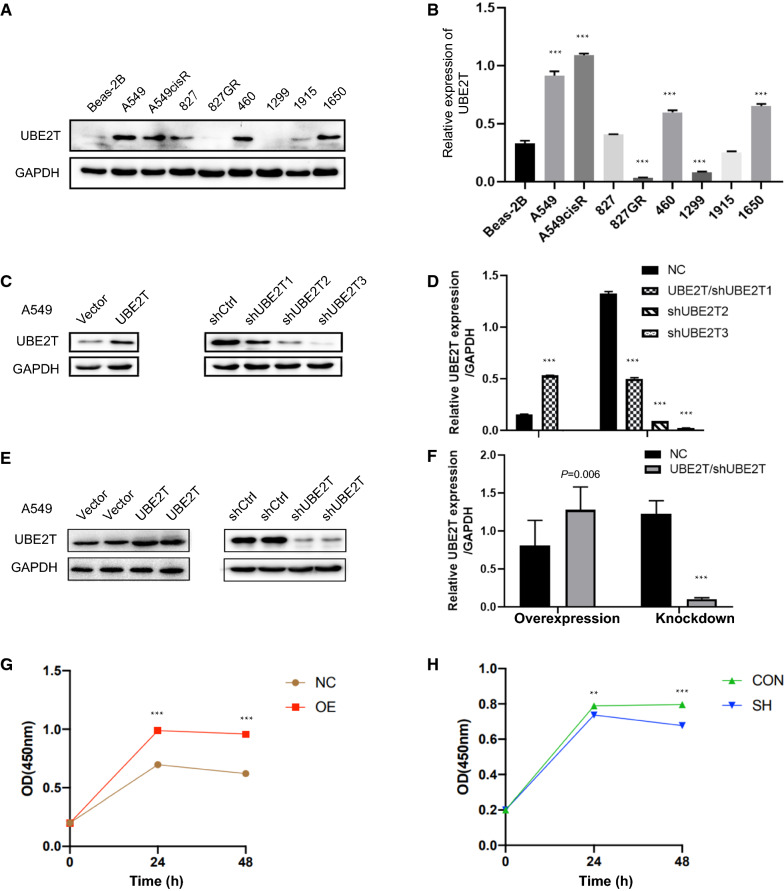
Elevated expression of UBE2T in most lung cancer cells. Western blot was used to detect UBE2T. **A**, **B** Comparison of UBE2T expression in normal lung bronchial epithelial and lung cancer cells. **C**, **D** UBE2T was overexpressed in A549 cells infected with a lentiviral vector carrying UBE2T cDNA. Differential gene silencing efficiency in A549 cells by three lentivirus-mediated short-hairpin RNAs designed against UBE2T (shUBE2T). **E**, **F** Establishment and validation of stable A549 cells with knockdown of UBE2T or overexpressing UBE2T. Western blot was conducted at least three times to validate the stable cells. GAPDH was used as internal controls. **G** UBE2T overexpression promoted the proliferation of A549 cells. **H** Silencing of UBE2T showed inhibitory effects on cell proliferation. All experiments were repeated at least three times. Protein bands were quantified using Image J. *P < 0.05, **P < 0.01, ***P < 0.001

Previous studies have demonstrated that UBE2T is overexpressed in lung cancer. In this study, we first examined the expression levels of UBE2T in eight lung cancer cell lines and a normal human lung epithelial cell line. Equal amounts of cell lysis were analyzed by western blot. The results showed that UBE2T expression was elevated in the majority of cancer cell lines compared with the normal lung epithelial cell line (Fig. 1A, B). The A549 cell line was chosen to perform a loss-of-function or gain-of-function study because of its high susceptibility to lentivirus infection. Lentivirus-mediated UBE2T-specific shRNA UBE2T or cDNA was adopted to knockdown s or overexpress UBE2T in A549 cells. The silencing efficiency and overexpression were confirmed using western blotting analysis (Fig. 1C, D). The shRNA with the highest knocking down efficiency was used for the subsequent experiment and marked as shUBE2T. Stable cell lines were established to explore the biological function of UBE2T, and expression levels of UBE2T were confirmed (Fig. 1D, E). We also observed that UBE2T overexpression enhanced cell proliferation, while knockdown of this gene inhibited the proliferation of A549 cells (Fig. 1G, H).

### UBE2T promoted autophagy in NSCLC cells

**Fig. 2:**
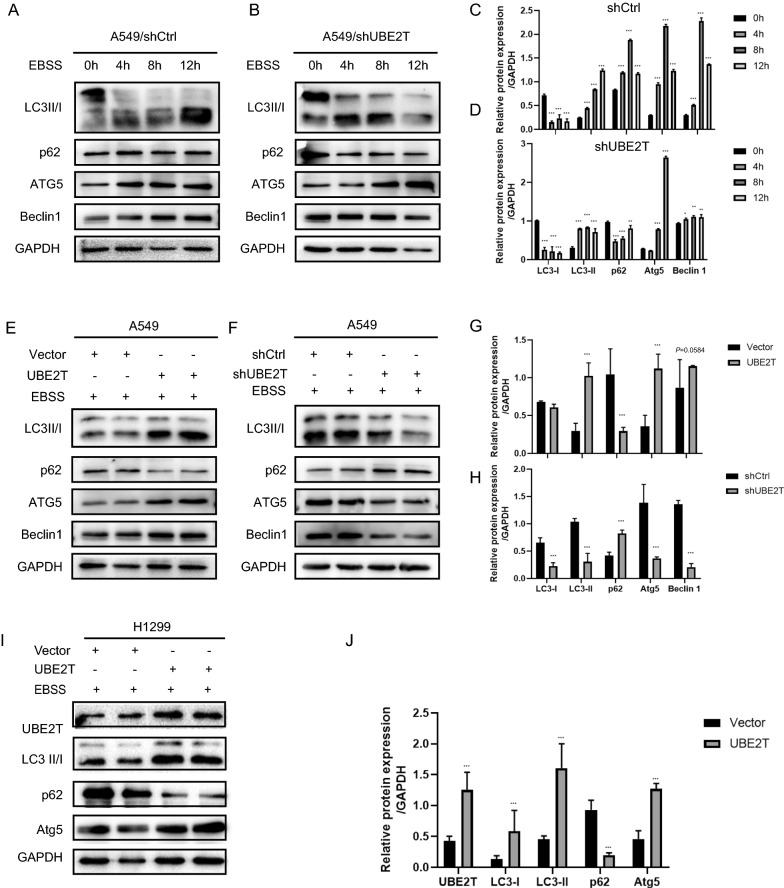
UBE2T upregulates autophagy in NSCLC cells. **A**–**D** Detection of autophagy in the labeled cells under starvation at different time points. Cells were starved in EBSS for 8 h to induce autophagy prior to analysis. Cell lysis was collected, and western blot was used to detect markers of autophagy. **E**, **G** Overexpression of UBE2T stimulated autophagy. **F**–**H** Silencing of UBE2T inhibited autophagy. **I**, **J** UBE2T overexpression increased autophagy in H1299 cells. GAPDH was used as internal controls. All experiments were repeated at least three times. Protein bands were quantified using Image J. *P < 0.05, **P < 0.01, ***P < 0.001

In physiological conditions, only basal levels of autophagy occur to maintain homeostasis in cells. However, autophagy will be triggered when cells are subjected to stresses, such as starvation [23], hypoxia [24] and, cytotoxic therapy. Autophagy can be evaluated by monitoring alterations in the expression of many critical autophagy-related proteins. The commonly used autophagy markers include, but are not limited to, LC3I, LC3II, P62, ATG5, and Beclin 1. LC3, Beclin-1, and ATG5 play essential roles in autophagosome formation, especially LC3-II levels directly proportional to autophagosome formation [25]. P62 is commonly used to reflect autophagic flux. In this study, we employed a starvation-induced in vitro autophagy model. Our results indicated that UBE2T upregulated autophagy in NSCLC cells. (Fig. 2A–D). As indicated in Fig. 2E, G, when compared to control cells, UBE2T overexpression increased levels of LC3II, ATG5, and Beclin 1 but decreased p62 levels in NSCLC cells after starvation, suggesting activation of autophagy. In the cells with UBE2T depletion, starvation-induced autophagy was attenuated (Fig. 2F, H). Besides, using 1299 cells with low endogenous UBE2T, we further confirmed that UBE2T overexpression increased autophagy (Fig. 2I, J).

### UBE2T regulated autophagy via altering nucleo-cytoplasmic localization of p53

**Fig. 3:**
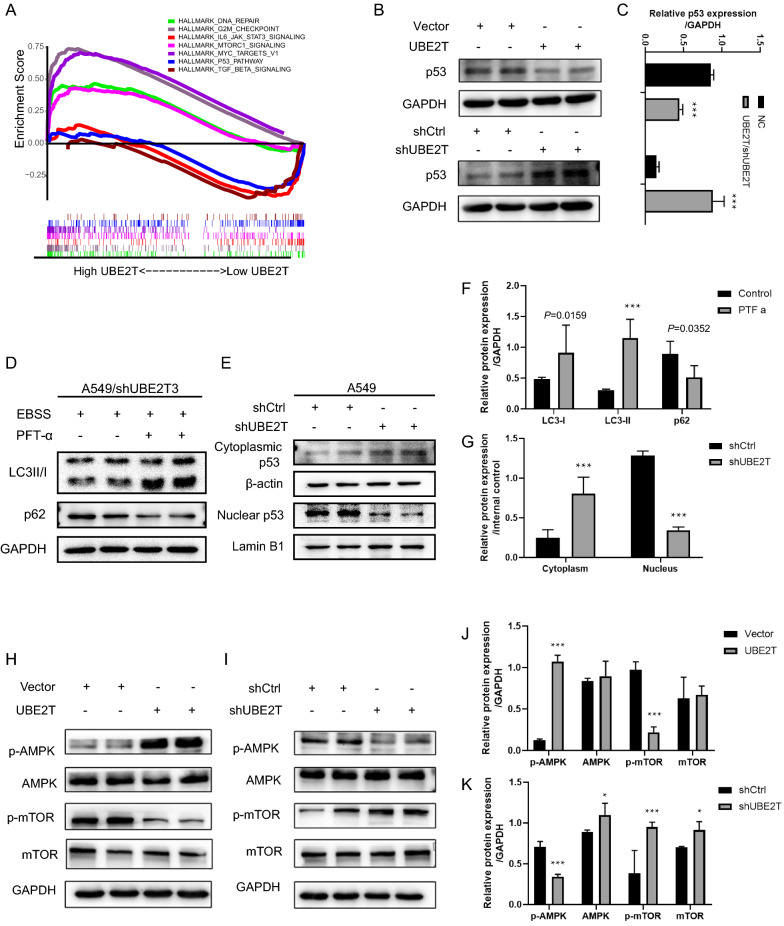
knockdown of UBE2T reduced autophagy by upregulating the expression of p53 and affecting its nuclear-cytoplasmic localization. **A** GSEA revealed the signaling pathways associated with UBE2T in NSCLC. **B**, **C** p53 expression levels were detected in UBE2T-deficient cells, UBE2T-overexpression cells, and the corresponding control cells via western blot analysis. **D**, **F** A549 cells infected with lentivirus-mediated shUBE2T were incubated in EBSS supplemented with or without pifithrin-a (PFT-a, 20 mM) for 8 h. After that, immunoblot analysis was performed with antibodies against LC3 and p62, separately. **E**, **G** The levels of nuclear p53 and cytoplasmic p53 in control cells and UBE2T deficient cells were examined after the separation of protein fractions using a cytoplasmic and nuclear extraction kit following the instruction. The effects of UBE2T on the AMPK/mTOR pathway were investigated. Cell lysis was subjected to western blot analysis with antibodies against p-AMPK, AMPK, p-mTOR, mTOR, and GAPDH. **H**, **J** UBE2T overexpression activated the AMPK/mTOR signaling pathway. **I**, **K** Silencing UBE2T inhibited the AMPK/mTOR signaling pathway. GAPDH was used as internal controls. All experiments were repeated at least three times. Protein bands were quantified using Image J. *P < 0.05, **P < 0.01, ***P < 0.001

To explore the downstream signaling pathway of UBE2T, we performed GSEA on lung cancer cell lines downloaded from the CCLE database. GSEA results signified that UBE2T was significantly associated with the mTORC1 and p53 signaling pathway (Fig. 3A). Our western blot results verified that UBE2T overexpression suppressed p53 expression compared with control cells. In contrast, silencing UBE2T led to increased p53 levels (Fig. 3B and C). To further investigate whether knockdown of UBE2T inhibited autophagy by upregulating p53, we blocked p53 with pifithrin-a (PFT-a), a pharmacological p53 inhibitor. We found that compared with NSCLC cells with UBE2T depletion, the addition of pifithrin-a (PFT-a) to UBE2T deficient cells restored autophagy, as indicated by the increased LC3II and decreased p62 in cells manipulated by genetically silencing UBE2T and p53 inhibitor (Fig. 3D, F). These results indicate that p53 may mediate UBE2T-regulated autophagy in A549 cells. Recent studies have revealed that p53 could either down- or upregulating autophagy depending on its subcellular localization [26]. Nuclear p53 facilitates autophagy by transactivating its target genes [27], whereas cytoplasmic p53 mainly inhibits autophagy [28]. With this in mind, we interrogated whether UBE2T affects autophagy by controlling cytonuclear trafficking of p53. After separating cytoplasmic and nuclear fractions of cells using the cytoplasmic-nuclear extraction kit, we found that knockdown of UBE2T caused cytoplasmic retention of p53, but the accumulation of p53 in nuclei was decreased when compared with the control cells (Fig. 3E, G). Based on the above results, it is reasonable to speculate that UBE2T regulates the level of autophagy, partially by influencing p53 nucleo-cytoplasmic shuttling.

### UBE2T promotes autophagy through p53/AMPK/mTOR pathway

Literature review unveiled that both wild-type or cytoplasm-localized p53 variants inhibited autophagy by reducing phosphorylation of AMPK and increasing phosphorylation of p70^S6k^, a putative substrate of mTOR [28]. Because we found that p53 mediated UBE2T-regulated autophagy, we, therefore, examine the effect of UBE2T on the AMPK/mTOR axis. In the current study, we found that UBE2T overexpression enhanced phosphorylation of AMPK but decreased phosphorylation of mTOR (Fig. 3H, J), and opposite results were observed in the UBE2T deficient A549 cells (Fig. 3I, K). Taken together, it seemed that silencing UBE2T led to the accumulation of p53 in the cytoplasm, and cytoplasmic p53 might, in turn, inactivates AMPK-mTOR signaling and inhibits autophagy.

**Fig. 4:**
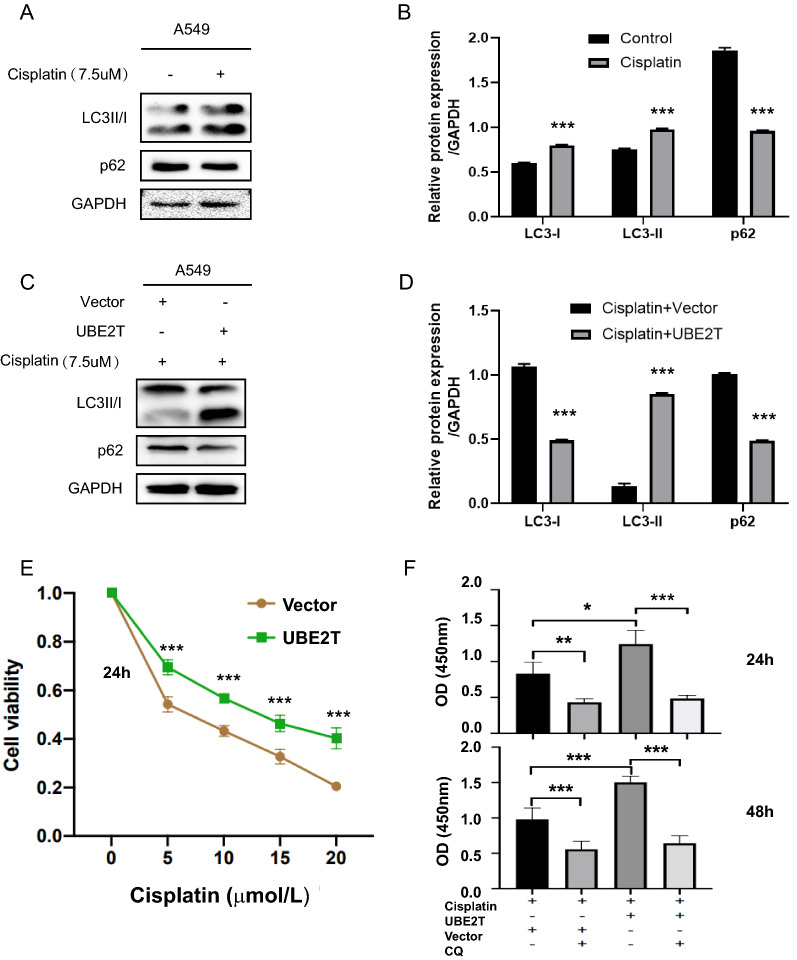
UBE2T augment cisplatin-induced autophagy. **A** Western blotting analysis was used to examine the effects of cisplatin treatments on autophagy in the A549 cells, followed by quantification using Image J (**B**). **C** Cisplatin-induced autophagy was further enhanced in the UBE2T overexpressing A549 cells, followed by quantification using Image J **D**. GAPDH was used as internal controls. **E** UBE2T overexpression decreased the sensitivity of A549 cells to cisplatin. **F** Autophagy inhibitor increased A549 cell’s sensitivity to cisplatin. All experiments were repeated at least three times. *P < 0.05, **P < 0.01, ***P < 0.001

Moreover, chemotherapy often elicits autophagy, which protects the cell from the harm of cytotoxic drugs. However, the mechanisms underlying the drug-induced protective autophagy are not clear. We found that cisplatin stimulated autophagy in A549 cells (Fig. 4A, B), and overexpression of UBE2T further increased autophagy levels, suggesting that UBE2T may be involved in the cisplatin-induced protective autophagy (Fig. 4C, D). Moreover, UBE2T overexpression ablated the sensitivity of A549 cells to cisplatin treatment (Fig. 4E) while inhibiting autophagy by chloroquine (CQ) reversed UBE2T-induced cisplatin resistance (Fig. 4F).

### Prognostic value of UBE2T in NSCLC

**Fig. 5:**
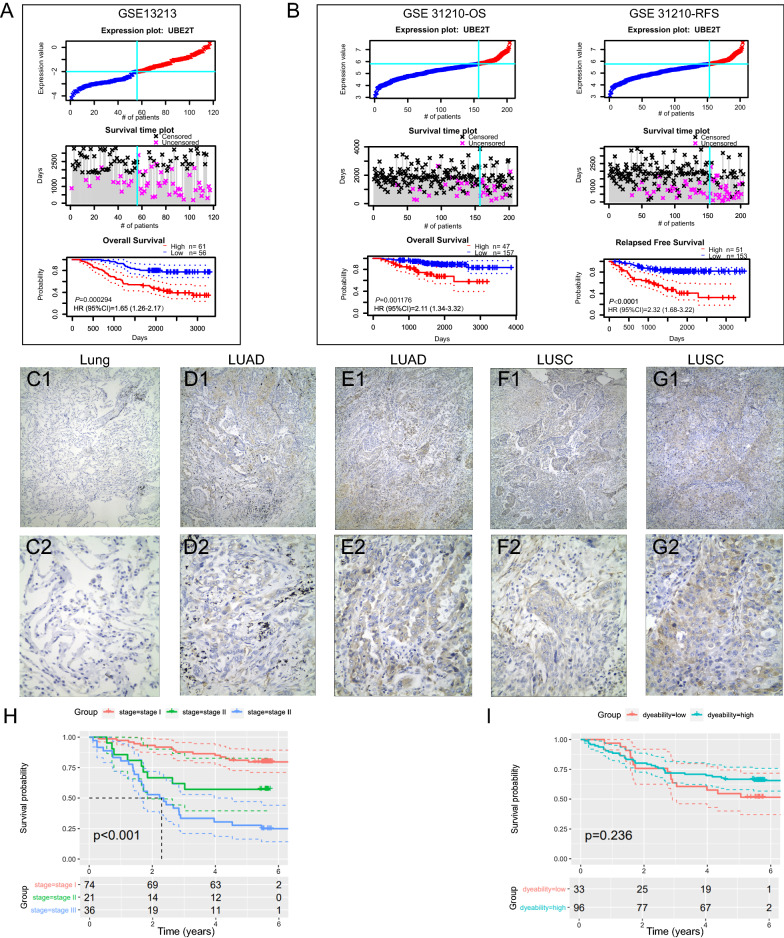
Prognostic value of UBE2T in NSCLC. **A** Kaplan–Meier survival curves presenting overall survival (OS) times for groups divided by the optimal cutoff value of *UBE2T* transcripts in GSE13213 (n = 117). **B** Recurrence-free survival (RFS) and OS for patients with *UBE2T*^high^ and *UBE2T*^low^ tumor in GSE31210 (n = 205). Immunohistochemical staining of UBE2T in NSCLC tissues (**C**–**G**). UBE2T staining in normal lung tissues (C1 100×, C2 400×); Low (D1 100×, D2 400×) and high (E1 100×, E2 400×) expression of UBE2T in LUAD; Low (F1 100×, F2 400×) and high (G1 100×, G2 400×) expression of UBE2T in LUSC. **H** Kaplan–Meier survival plots for TNM stages. **I** Kaplan–Meier survival plots for immunostaining scores of UBE2T

A previous study demonstrated that high UBE2T levels were associated with unfavorable prognosis in the LUAD cohort of The Cancer Genome Atlas project [17]. Here, we validated the prognostic value of UBE2T in two GEO LUAD cohorts, GSE13213 (n = 117) and GSE31210 (n = 205). Patients with *UBE2T*^low^ tumors significantly outperformed those with *UBE2T*^high^ tumors in survival in these two LUAD cohorts (Fig. 5A, B). We evaluated the prognostic accuracy of UBE2T in the TCGA-LUAD cohort using ROC curves. Unfortunately, the ROC value of UBE2T was even smaller than that of the TNM stage (0.561 vs. 0. 655, data not shown), suggesting a poor predictive value of UBE2T. T, N, and M represent the size or direct extent of the primary tumor, degree of spread to regional lymph nodes, and presence of distant metastasis, respectively.

We tried to validate the prognostic value of UBE2T in the NSCLC patients. We enrolled 131 patients with resectable NSCLC. Among them, we successfully conducted UBE2T immunohistochemical staining in 129 samples. The UBE2T immunoreactivity was mainly seen in the cytoplasm of tumor cells (Fig. 5C–G). According to staining intensity, the UBE2T expression was scored from 0 to 3. We classified NSCLC tissues with immunostaining scores ≤ 1 or ≥ 2 into UBE2T^low^ and UBE2T^high^ groups, respectively. The association of the clinical-stage with overall survival (OS) of NSCLC patients was shown in Fig. 5H, but UBE2T protein levels were not associated with OS of ESCC patients (Fig. 5I). These results suggest that UBE2T alone may not be adequate to predict prognosis in NSCLC, emphasizing the importance of the multi-gene signatures in predicting prognosis.

### Prognostic value of UBE2T in combination with autophagy genes in LUAD

**Fig. 6:**
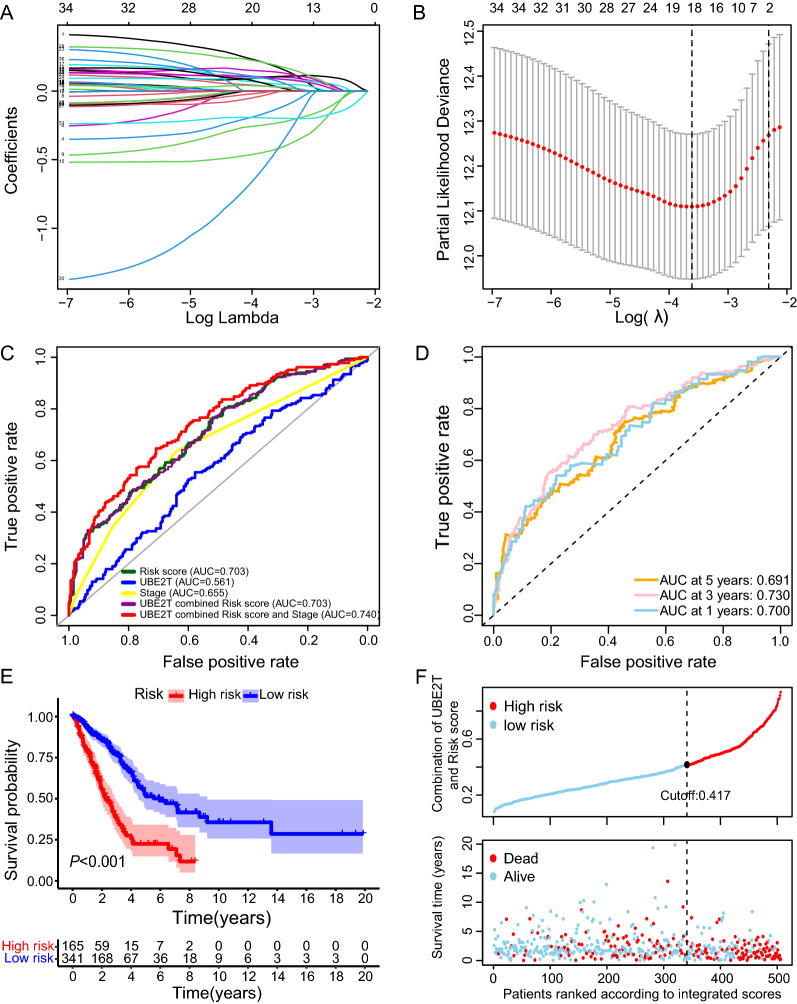
The prognostic accuracy of the risk estimation model derived from *UBE2T*-related autophagy genes in the TCGA-LUAD cohort. To improve the prognostic accuracy of *UBE2T*, the LASSO regression model was used to establish the best risk signature from the *UBE2T*-correlated autophagy genes in the TCGA-LUAD cohort. **A** Lasso coefficients of *UBE2T*-related autophagy genes. **B** Determination of the optimal gene signature in the LASSO model. **C** 5-year ROC curves for indicated variables. The risk score has the highest AUC value of all individual risk factors. **D** Time-dependent ROC curves for the risk score. The cutoff value of the risk scores was evaluated by plotting ROC curves. **E** LUAD patients were divided into low- and high-risk groups by the cutoff value. The risk score is associated with patient OS. The Kaplan–Meier survival curves of OS time between low- and high-risk patients. **F** The distribution of the integrated score and survival status of the TCGA-LUAD patients

**Fig. 7:**
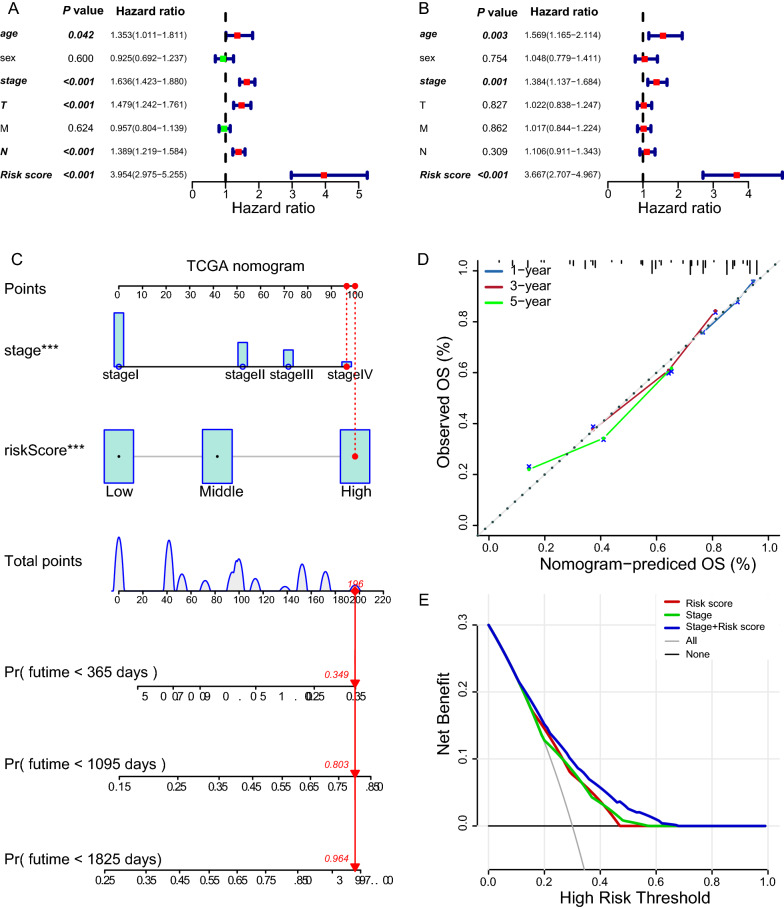
Discriminatory performance of the risk score, TNM stage, and in combination in the TCGA-LUAD cohort. **A** Forest plot of univariate Cox regression analyses. **B** Forest plot of multivariate Cox regression analyses. **C** The nomogram for predicting individual OS possibility. **D** The calibration plots for the prediction of 1-, 3- and 5-year survival. The x-axis and y-axis denote nomogram-predicted (solid line) and actual survival, respectively, with the vertical bars representing a 95% confidence interval. **E** Decision curve analysis of indicated variables across probability thresholds. The combined model of the risk score and TNM stage has the highest net benefit at any given threshold

We then tried to improve the prognostic accuracy of UBE2T by considering its biological function. Given the critical role of UBE2T in regulating autophagy, we first obtained 163 autophagy-related genes that closely correlated with UBE2T, followed by univariate Cox analysis screening for survival genes. We entered 36 autophagy-related survival genes into a LASSO regression model and generated a signature of 18 autophagy-related survival genes (Fig. 6A, B). Next, we employed a risk score algorithm to evaluate the patient risk for overall survival by integrating the expression levels of each included gene with its survival coefficient. As shown in Fig. 6C, the ROC of the resulting risk score was 0.703, indicating that the risk score performance exceeded UBE2T alone. While coupled with TNM and UBE2T, the performance of the risk score culminated as revealed by a ROC value of 0.740 (Fig. 6C, D). Kaplan–Meier survival curves and Cox regression analyses demonstrated that the UBE2T-correlated autophagy gene signature was significantly associated with survival and was an independent predictor of prognosis in LUAD (Figs. 6E, F; 7A, B). We also combined the risk score with TNM to build a nomogram to predict patients’ survival possibility, achieving a *C*-index of 0.714 (Fig. 7C, D). Decision curve analysis illustrated that the composition of the risk score and TNM showed a better net benefit than either variable alone (Fig. 7E).

### Prediction of sensitivity to cancer therapy

**Fig. 8:**
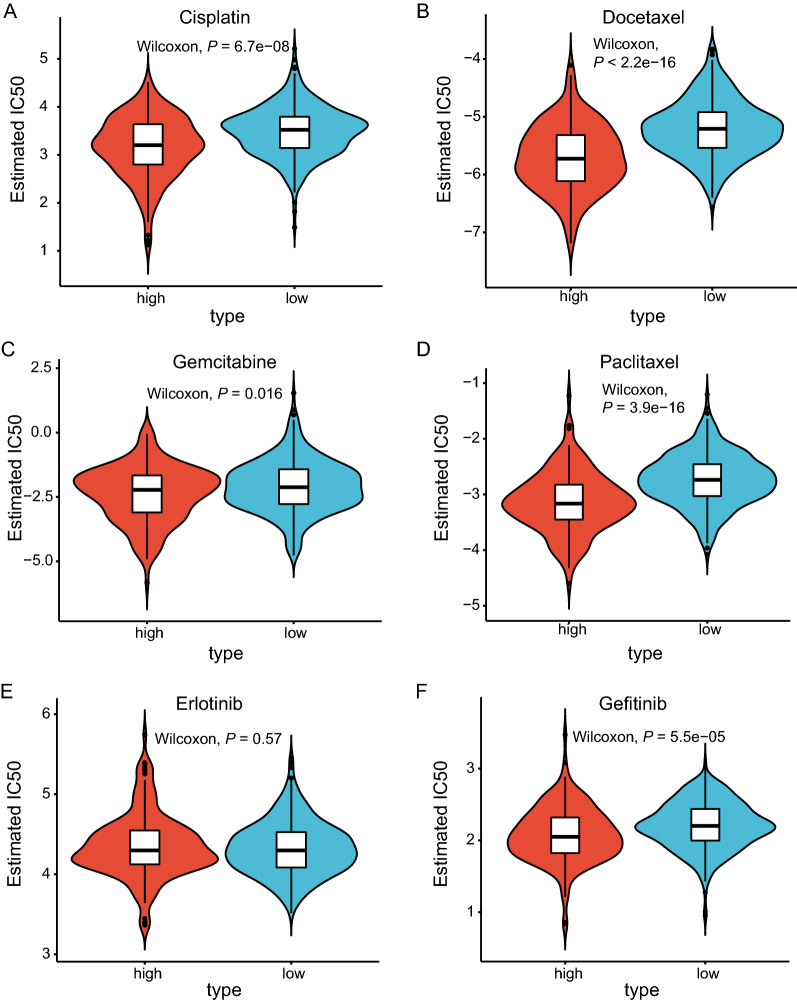
Prediction of chemosensitivity in the TCGA-LUAD cohort using the prognostic signature. Comparison of IC50 of common chemotherapeutic drugs between high- and high-risk groups, including cisplatin (**A**), docetaxel (**B**), gemcitabine (**C**), paclitaxel (**D**), erlotinib (**E**), and gefitinib (**F**)

We also tested whether the risk score can predict clinical drug response. Cisplatin, docetaxel, gemcitabine, paclitaxel, erlotinib, and gefitinib are frequently used to treat lung cancer patients. It has been extremely challenging to discriminate patients suitable for these therapies. As a result, only a proportion of patients can really benefit from routine cancer therapy. Physicians desperately call reliable biomarkers for better prediction of therapeutic benefit. Fortunately, recent progress in next-generation sequencing and the pRRophetic methods permit us to predict clinical chemotherapeutic response based on gene expression levels. As indicated by IC50, the high-risk group was significantly more likely to respond to cisplatin, docetaxel, gemcitabine, paclitaxel, and gefitinib (Fig. 8A–F). These results indicated that this risk score has great translational potentials predictive of prognosis and drug sensitivity.

## Discussion

NSCLC is leading cancer and also one of the deadliest malignant tumors in the world. Irrespective of the great progress made in multimodality, including surgery, chemotherapy, radiotherapy, and targeted therapy, the 5-year overall survival of NSCLC remains disappointing. NSCLC is a complicated multifaced disease. What we know about NSCLC to date is just the tip of the iceberg by far. Intensive and continuous effects are warranted to clarify the potential mechanisms involved in tumorigenesis of NSCLC.

It is becoming clear that the role of autophagy is highly context-dependent in the development of cancer. In particular, autophagy plays a prosurvival role in established tumors. Tumor cells, which are often exposed to metabolic and hypoxic stress, severely rely on autophagy to supply nutrients by reprocessing cargos and producing biomacromolecules. Several lines of evidence illustrate the implication of autophagy in lung cancer. For instance, inhibiting autophagy by genetical deletion of *ATG7,* an essential autophagy gene, suppressed tumor growth in the KRAS^G12D^-driven NSCLC mouse model [29]. ATG10, an autophagy-related protein, boosted lung cancer cell proliferation and migration [30]. The same study further denoted that the enhanced expression of ATG10 might associate with shortened survival time in lung cancer [30]. We previously showed that a signature of 22 autophagy-related genes significantly stratified LUAD patients into high- and low-risk groups according to OS [19]. Similarly, another group reported that a prognostic signature consisting of 6 autophagy-related genes (EIF4EBP1, TP63, BNIP3, ATIC, ERO1A, and FADD) could be used to predict survival in NSCLC [31]. The studies provided strong evidence of the association between autophagy and lung cancer. However, the deep mechanisms behind the contributions of autophagy to carcinogenesis still warrant further investigation.

In this study, we found that UBE2T was upregulated in most tested lung cancer cell lines when compared with control cells. Interestingly, UBE2T enhanced autophagy in NSCLC cells under starvation. The oncogenic contribution of UBE2T has been manifested in a wide spectrum of cancers from solid tumors to multiple myeloma [13]. Several studies have suggested the important implications of UBE2T in NSCLC [15–17]. UBE2T is significantly associated with poor prognosis in lung carcinoma [16, 17]. Moreover, UBE2T was found to stimulate NSCLC cell proliferation, migration, invasion, and epithelial-mesenchymal transition (EMT) [15]. The roles of UBE2T in NSCLC carcinogenesis were mostly unknown. This study presents a novel mechanism that UBE2T may foster the development of NSCLC by stimulating autophagy.

Autophagy is a highly and accurately regulated cellular process. Several signaling pathways have been reported to play a crucial role in regulating autophagy, including the p53, PI3K, RAS, and JAK-STAT signaling pathways [6]. While interrogating how UBE2T evokes autophagy in NSCLC cells, we found that UBE2T, which stimulated autophagy, downregulated p53 in A549 cells. On the other hand, silencing UBE2T decrease autophagy, along with an increase in p53. Blockade of p53 abolished the autophagy-inhibitory effect of UBE2T depletion. These results indicate that UBE2T increases autophagy by inhibiting p53 in NSCLC cells. The function of p53 is partially controlled by nuclear-cytoplasmic shuttling [32]. This factor makes the mechanisms of p53 dependent autophagy intriguing and complex. Recent studies reveal that p53 may either down- or upregulating autophagy depending on its subcellular localization [26]. Nuclear p53 facilitates autophagy by transactivating its target genes [27], whereas cytoplasmic p53 mainly inhibits autophagy [28]. To date, two types of mechanisms underlying p53-regulated autophagy are reported, including transcription-independent (e.g., AMPK activation) [28] and transcription-dependent functions (e.g., transactivation of PTEN and TSC1, or damage-regulated autophagy modulator, DRAM) that inhibit mTOR activity [27, 33, 34]. Our results indicated that UBE2T deficiency caused cytoplasmic retention of p53 in the NSCLC cells, concomitant with decreased autophagy levels. Consistently. Our results are in agreement with others. Tasdemir and collegues observed that a p53 variant preferentially localized in the cytoplasm suppressed autophagy, but not the p53 variant destinated to the nucleus [28]. Moreover, colistin was found to induce autophagy through regulating nuclear-cytoplasmic shuttling of p53 in rat adrenal medulla PC-12 Cells [35]. Collectively, it is reasonable to speculate that silencing UBE2T inhibits autophagy by increasing cytoplasmic p53.

The AMPK/mTOR signaling pathway is a vital regulation pathway of cellular autophagy [36]. Autophagy may occur upon different stress stimuli, such as starvation, hypoxia, endoplasmic reticulum (ER) stress, and oxidative stress [25]. Even under these different conditions, autophagy is often a result of activation of the nutrient energy sensor AMP kinase (AMPK) and inactivation of mTOR (target of rapamycin) [33]. In the AMPK/mTOR signaling pathway, AMPK is activated by phosphorylation, which and then phosphorylates and inhibits mTOR, ultimately triggering autophagy [36]. Consistent with the finding that cytoplasmic p53 downregulated autophagy by inactivating the AMPK/mTOR pathway [28], our results validated when silencing UBE2T caused cytoplasmic accumulation of p53, the activity of the AMPK/mTOR signaling pathway was diminished. Overall, we found that UBE2T promotes autophagy through the p53/AMPK/mTOR signaling pathway in NSCLC.

Because it is urgent to discover novel biomarkers for cancer prognosis to guide effectively personalized therapeutic intervention, we also evaluated the prognostic value of UEB2T. We found that UBE2T was associated with poor survival in two independent GEO-LUAD cohorts. These results are consistent with others’ findings in the TCGA-LUAD cohort [16]. However, we observed that the UBE2T gene alone showed inferior sensitivity and specificity in predicting prognosis in the TCGA-LUAD cohort.

It is not surprising that current individual gene biomarkers often fail to predict cancer prognosis accurately because a gene can hardly capture the distinct molecular heterogeneity of cancer. This challenge has spurred the development of multiple gene signatures predictive of outcomes in a broad spectrum of cancer [20, 37]. Gene expression profile-based prognostic risk scores have recently emerged as promising biomarkers with substantial clinical values. Compared to single biomarkers or traditional tumor staging, these composite multi-gene models are superior in identifying high-risk patients or predicting chemotherapy response. To improve the prognostic accuracy of UBE2T, we developed a prognostic signature with 18 autophagy-related genes correlated with UBE2T. The combination of UBE2T and the risk signature significantly improved prognosis prediction compared with the UBE2T gene alone. The prognostic accuracy was validated by a nomogram and DCA. Besides, the prognostic signature was also associated with sensitivity to chemotherapy. Taken together, these results show that UBE2T and its autophagy regulating role play critical roles in the development of LUAD.

Several limitations were as follows. First, most of the findings were derived from western blotting results. Second, the NSCLC sample size was relatively small. Third, a p53 mutant (using a disrupted nuclear export signal, NES) restricted to nuclei should be used to confirm that silencing of UBE2T suppressed autophagy by promoting cytoplasmic localization of p53.

In summary, our study demonstrated that UBE2T promotes autophagy in NSCLC cells by modulating the p53/AMPK/mTOR signaling pathway. We validated the association of UBE2T and OS in LUAD. Moreover, we improved the ability of UBE2T to predict the prognosis and drug response in LUAD by building a robust signature with considering the autophagy-regulatory role of UBE2T.

## Supplementary Information

**Additional file 1: Table S1.** Charateristics of NSCLC patients.

## Data Availability

The datasets used and/or analyzed during the current study are available from the corresponding author on reasonable request.
