# Supplementary material for: UBE2T promotes autophagy via the p53/AMPK/mTOR signaling pathway in lung adenocarcinoma
Source: J Transl Med. 2021 Aug 30;19:374. doi: 10.1186/s12967-021-03056-1 (PMC8407090; doi:10.1186/s12967-021-03056-1)
Supplement: Supplementary file 1 — Additional file 1: Table S1. Charateristics of NSCLC patients. [file 12967_2021_3056_MOESM1_ESM.docx]

| Table S1. Charateristics of NSCLC patients. | |
| --- | --- |
|  | **Overall** |
| **N** | 131 |
| **Pathology (%)** |  |
| **LUAD** | 72 ( 55.0 %) |
| **LUSC** | 52 ( 39.7 %) |
| **Other** | 7 ( 5.3 %) |
| **Survival state (%)** |  |
| **Alive** | 80 ( 61.1 %) |
| **Dead** | 51 ( 38.9 %) |
| **Stage (%)** |  |
| **Stage I** | 74 ( 56.5 %) |
| **Stage II** | 21 ( 16.0 %) |
| **Stage III** | 36 ( 27.5 %) |
| **T (%)** |  |
| **T1** | 58 ( 44.3 %) |
| **T2** | 65 ( 49.6 %) |
| **T3** | 5 ( 3.8 %) |
| **T4** | 3 ( 2.3 %) |
| **N (%)** |  |
| **N0** | 80 ( 61.1 %) |
| **N1** | 14 ( 10.7 %) |
| **N2** | 37 ( 28.2 %) |
| **M (%)** |  |
| **M0** | 131 (100.0 %) |
